# Влияние дооперационной терапии бисфосфонатами на минеральную плотность костной ткани у пациентов с ПГПТ через год после паратиреоидэктомии

**DOI:** 10.14341/probl13574

**Published:** 2025-09-14

**Authors:** А. П. Першина-Милютина, А. К. Еремкина, И. Д. Ожималов, А. В. Хайриева, А. М. Горбачева, С. В. Ронжина, Н. Г. Мокрышева

**Affiliations:** Национальный медицинский исследовательский центр эндокринологии им. академика И.И. Дедова; I.I. Dedov Endocrinology Research Centre; Московский государственный университет им. М.В. Ломоносова; Lomonosov Moscow State University; Первый Московский государственный медицинский университет им. И.М. Сеченова (Сеченовский университет); I.M. Sechenov First Moscow State Medical University

**Keywords:** первичный гиперпаратиреоз, DXA, 3D-DXA, бисфосфонаты, паратиреоидэктомия, primary hyperparathyroidism, DXA, 3D-DXA, bisphosphonates, parathyroidectomy

## Abstract

**BACKGROUND:**

BACKGROUND: The main treatment for primary hyperparathyroidism (PHPT) is parathyroidectomy (PTE), conservative therapy, including bisphosphonates, can be used for preoperative correction of hypercalcemia, as well as to improve bone tissue condition among individuals for whom surgery should be postponed or cannot be performed due to high perioperative risks. The question of the effect of bisphosphonates on bone tissue after surgery remains open.

**AIM:**

AIM: To study the effect of preoperative bisphosphonate therapy on BMD parameters assessed in DXA and 3D-DXA in patients with PHPT one year after radical PTE.

**MATERIALS AND METHODS:**

MATERIALS AND METHODS: The study was conducted on the basis of the Department of pathology of the parathyroid glands and disorders of mineral metabolism of "Endocrinology Research Center" state-funded research facility of the Ministry of Health of the Russian Federation. The study included 50 patients (2 men, 48 women), divided into two groups depending on the presence or absence of preoperative bisphosphonate (BF) therapy. The methods of DXA and 3D-DXA using 3D-Shaper Medical software were used to evaluate BMD and bone microarchitectonics. The statistical analysis was performed using the R language and the Statistica v.13 package.

**RESULTS:**

RESULTS: At the time of the disease’s manifestation, both groups were comparable in terms of the main indicators of calcium phosphorus metabolism, with the exception of the level of beta-crosslapse, which was higher in the group without preoperative BPh therapy (p<0,001). There were also no differences in the parameters of DXA and 3D-DXA. After surgery, both groups showed a comparable increase in BMD based on the results of DXA in the main parts of the skeleton and 3D-DXA in the femur. Changes at the level of the statistical trend were obtained for the 3D-DXA parameters, the final absolute values of which were slightly higher in the second group, including the thickness of the cortical layer in the femur as a whole and in the neck. When comparing the results of DXA before and after PTE in patients receiving BPh, statistically significant differences in absolute BMD values were obtained only in the lumbar spine (p<0,001).According to 3D-DXA data, statistically significant differences were found only in the volume of mineral density of the trabecular bone of the femur as a whole (p=0,001).

When analyzing up to — in the second group, statistically significant differences in absolute BMD values were observed in the lumbar region (p<0,001), in the hip as a whole (p<0,001) and in its neck (p=0,001).According to 3D-DXA data, statistically significant differences were found in three of the eight analyzed indicators, the volume of mineral density of the trabecular bone of the femur as a whole and in the neck (p<0,001 for both), as well as the volume of mineral density of the cortical bone in the neck, (p=0,001).

**CONCLUSION:**

CONCLUSION: The 3D-DXA method allows us to evaluate not only BMD, but also its microarchitectonics, which is important for predicting the risk of fractures in patients with PHPT. Studies have shown that preoperative BPh therapy can negatively affect the recovery of BMD after PTE, especially in cortical bone tissue. Further studies are needed to confirm these data and clarify the effect of CF on the postoperative course of PHPT.

## ОБОСНОВАНИЕ

Первичный гиперпаратиреоз (ПГПТ) — эндокринное заболевание, возникающее вследствие гиперпродукции паратиреоидного гормона (ПТГ) патологически измененными околощитовидными железами (ОЩЖ), как правило, сопровождающееся повышением концентрации кальция крови. Частота ПГПТ в общей популяции составляет в среднем 0,86–1% [1]: от 0,1 (США) до 0,43% (Швеция) [2]. В российской популяции истинная распространенность ПГПТ неизвестна, что прежде всего связано с отсутствием рутинного определения кальция в сыворотке крови [3].

Избыточная концентрация ПТГ в крови приводит к активации остеокластов и интенсификации процессов костной резорбции, поэтому наиболее частым осложнением ПГПТ является снижение минеральной плотности костной ткани (МПК) вплоть до остеопороза. Наиболее распространенный метод диагностики остеопороза — двухэнергетическая рентгеновская абсорбциометрия (DXA). Определяемая при проведении DХА МПК является критерием для постановки диагноза «остеопороз». Снижение МПК по Т-критерию ≤-2,5 SD для женщин после наступления менопаузы и мужчин старше 50 лет или по Z-критерию ≤-2,0 SD для женщин до наступления менопаузы и мужчин моложе 50 лет, или наличие низкоэнергетического перелома (перелом проксимального эпифиза бедренной кости, компрессионные переломы тел позвонков) считаются основаниями для постановки диагноза. Существуют и другие методы оценки МПК, основанные на компьютерной томографии, ультразвуковом исследовании и проч., но одним из наиболее перспективных является метод 3D-моделирования костной ткани (3D-DХА), способный по данным, полученным с помощью обычной DХА, построить трехмерную реконструкцию бедренной кости и определить объемные характеристики МПК. Работы с применением данной технологии в когорте пациентов с ПГПТ пока ограничены.

В российской популяции у 62,5% пациентов с ПГПТ диагностируются костные осложнения заболевания [4], что сопровождается существенным снижением качества жизни, увеличением инвалидизации и смертности. Единственным патогенетическим лечением ПГПТ остается паратиреоидэктомия (ПТЭ) [5]. При невозможности проведения ПТЭ допустимо консервативное лечение, направленное на замедление процессов костной резорбции и снижение кальциемии. Одной из групп препаратов, применяемых в таких случаях, являются бисфосфонаты (БФ). Их особенностью является фармакокинетика — БФ имеют высокую тропность к костной ткани, поэтому период их полувыведения может достигать нескольких месяцев и даже лет [6].

Хотя радикальная ПТЭ приводит к нормализации уровня ПТГ, восстановление костной ткани занимает значительное время. В соответствии с российскими клиническими рекомендациями, в течение года после ПТЭ назначать антирезорбтивную терапию не рекомендуется. С одной стороны, это может усугубить послеоперационную гипокальциемию и способствовать вторичному гиперпаратиреозу. С другой, имеются данные о потенциальном негативном влиянии БФ на прирост МПК после операции по сравнению с пациентами без терапии [5][7]. Нерешенным остается вопрос о пролонгации терапии у пациентов, которые получали БФ до операции. Единого экспертного мнения по этому вопросу на сегодняшний день нет.

## ЦЕЛЬ ИССЛЕДОВАНИЯ

Изучить влияние дооперационной терапии бисфосфонатами на параметры МПК, оцениваемые при DXA и 3D-DXA, у пациентов с ПГПТ через год после радикальной ПТЭ.

## МАТЕРИАЛЫ И МЕТОДЫ

## Место и время проведения исследования

Место проведения. Исследование было проведено на базе отделения патологии околощитовидных желез и нарушений минерального обмена ФГБУ «НМИЦ эндокринологии» Минздрава России, г. Москва.

Время исследования. В исследование включены пациенты, госпитализированные в указанное отделение в период с октября 2016 по февраль 2024 гг.

## Изучаемые популяции (одна или несколько)

Критерии включения в исследование:

Критерии невключения: отсутствие информации о медикаментозной терапии до ПТЭ.

Дизайн исследования представлен на рисунке 1. Способ формирования выборки — сплошной.

**Figure:**
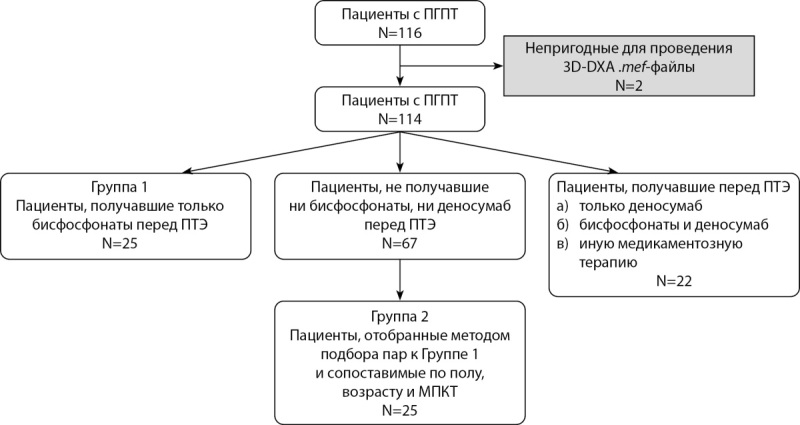
Рисунок 1. Дизайн формирования групп исследования.

В результате первичного отбора пациентов в исследуемую группу вошли 116 человек, которые далее были разбиты на 2 группы в зависимости от наличия или отсутствия дооперационной терапии БФ, где первая группа — пациенты, получающие терапию.

## Дизайн исследования

Настоящее исследование носит характер одноцентрового, обсервационного, ретроспективного, динамического (первая рентгенологическая денситометрия выполнена не ранее, чем за год до ПТЭ, вторая — не ранее, чем через год после проведения ПТЭ), сравнительного двухвыборочного.

## Методы

Все лабораторные исследования были выполнены на базе лаборатории ФГБУ «НМИЦ эндокринологии» Минздрава России и оценены в соответствии с локальными референсными значениями.

Оценивались следующие показатели: кальций общий, кальций ионизированный, альбумин, щелочная фосфатаза, суточная кальциурия, креатинин (на автоматическом биохимическом анализаторе ARCHITECH с8000 (Abbott, CША)), интактный ПТГ, СТХ, остеокальцин (на электрохемилюминесцентном анализаторе Cobas 6000 (Roche, Германия)), 1,25-(OH)-D (на анализаторе Liaison XL (DiaSorin, Италия)).

Скорость клубочковой фильтрации (СКФ) рассчитывалась по формуле CKD-EPI, скорректированный кальций — по формуле [Са](скорр.)=[Са](общ.)+0,8∙(4-Alb),

где

Са (скорр.) — концентрация кальция, скорректированного на альбумин (ммоль/л);

Са (общ.) — концентрация ионизированного кальция (ммоль/л);

Alb — концентрация альбумина (г/л).

Абсолютная МПК, Т- и Z-критерии определялись с помощью DXA в трех отделах скелета — в дистальном отделе лучевой кости, проксимальном отделе бедренной кости и поясничном отделе позвоночника (Lunar iDXA, GE Healthcare, Япония или Hologic Discovery, США).

Для выявления особенностей повреждения кортикального и трабекулярного компонента костной ткани использовалось новое программное обеспечение 3D-Shaper Medical (certified by BSI to ISO 13485:2016 under certificate number MD 731095; Version v2.12.1, Испания), способное создавать 3D-модели проксимального отдела бедренной кости пациента на основе метаданных, полученных после стандартного денситометрического исследования. Оценивались следующие параметры: минеральная плотность поверхности кортикальной кости (cortical surface bone mineral density — cortical sBMD; мг/см²); объем минеральной плотности кортикальной кости (cortical volumetric bone mineral density — cortical vBMD; мг/см³); толщина кортикальной кости (cortical thickness; мм); объем минеральной плотности трабекулярной кости (trabecular volumetric bone mineral density — trabecular vBMD; мг/см³).

Наличие компрессионных переломов тел позвонков устанавливалось при рентгенографии поясничного и грудного отделов позвоночника в боковой проекции (рентгенодиагностическая система Optima RF420, GE Healthcare, Япония).

Нефрокальциноз и нефролитиаз выявлялись при УЗИ почек и забрюшинного пространства (УЗИ-аппарат Aplio 500, Toshiba, Япония) или мультиспиральной КТ (компьютерный томограф GE Optima CT660, США).

ПТЭ всем пациентам была выполнена в Отделе хирургии ФГБУ «НМИЦ эндокринологии» Минздрава России. Морфологический диагноз (аденома, атипическая опухоль, карцинома, гиперплазия) устанавливался согласно классификации опухолей эндокринных органов ВОЗ 2017/2022 гг. на базе референс-центра патоморфологических и иммуногистохимических методов исследования. Радикальность ПТЭ оценивалась по нормализации или снижению концентрации ПТГ более чем на 50% в раннем послеоперационном периоде [5].

## Статистический анализ

Статистический анализ проведен с использованием статистического языка программирования R (v.4.2.2) и программного пакета Statistica v.13 (TIBCO Inc., США). Описательная статистика количественных показателей представлена медианами, первым и третьим квартилями в виде Me [ Q1; Q3], качественных показателей — абсолютными и относительными частотами в виде n (%). Сравнение двух независимых групп для количественных данных выполнялось с помощью критерия Манна-Уитни, зависимых — с помощью критерия Вилкоксона. Сравнение двух независимых групп для качественных признаков выполнялось с помощью критерия Фримена-Холтона и точного двустороннего критерия Фишера, в случае бинарных признаков. Критический уровень статистической значимости при проверке статистических гипотез принят равным 0,05. При множественных сравнениях применялась поправка Бонферрони путем коррекции критического уровня значимости. Рассчитанные уровни значимости в диапазоне от критического до 0,05 описаны в качестве индикаторов статистической тенденции.

## Этическая экспертиза

Протокол исследования одобрен локальным этическим комитетом ФГБУ «НМИЦ эндокринологии» Минздрава России (протокол №1 от 17.01.2018).

## РЕЗУЛЬТАТЫ

В исследование были включены 50 пациентов, среди них — 2 мужчин (4%) и 48 женщин (96%). Медиана возраста на момент госпитализации в группе 1 составила 59 [ 56; 62] лет, во второй — 60  [ 55; 64] лет. Обе группы были сопоставимы по полу, возрасту на момент госпитализации, а также степени снижения МПК до операции (таблица 1, 2). Подробная характеристика показателей кальций-фосфорного обмена представлена в таблице 1. При сравнении двух групп по дооперационным лабораторным показателям статистически значимые различия были обнаружены только по уровню бета-кросслапса, который был выше в группе без предоперационной терапии БФ (1,315 [ 1,010; 1,720] нг/мл против 0,531 [ 0,305; 0,976] нг/мл, p=0,0003). Значения фосфора, ЩФ и остеокальцина были выше во второй группе, однако оставаясь на уровне статистической тенденции (табл. 1).

**Table:** Таблица 1. Сравнительная характеристика исследуемых групп пациентов по анамнестическим данным и основным характеристикам ПГПТ Сокращения: НЭП — низкоэнергетический перелом; иПТГ — интактный паратгормон; рСКФ — расчетная скорость клубочковой фильтрации (CКФ-EPI). Примечания: 1 — критерий Манна-Уитни; 2 — двусторонний точный критерий Фишера.

Признак	Пациенты, принимающие бисфосфонаты перед операцией	Пациенты, не принимающие бисфосфонаты перед операцией	р
N	Me [ Q1; Q3] / n (%)	N	Me [ Q1; Q3] / n (%)
Возраст на момент госпитализации, лет	25	59 [ 56; 62]	25	60 [ 55; 64]	0,6618¹
Пол	Мужской	25	1 (4%)	25	1 (4%)	1,0000²
Женский	24 (96%)	24 (96%)
Вес, кг	25	67 [ 62; 74]	24	68,5 [ 61,5; 83]	-
Рост, см	25	158 [ 153; 164]	24	161,5 [ 157; 167]	-
ИМТ, кг/м²	25	26,22 [ 24,78; 30,02]	24	26,64 [ 23,84; 31,12]	-
Данные лабораторных исследований до операции
Са общий, ммоль/л	25	2,71 [ 2,61; 2,89]	25	2,77 [ 2,67; 2,86]	0,5475¹
Са ион., ммоль/л	17	1,26 [ 1,21; 1,35]	17	1,29 [ 1,25; 1,37]	0,2856¹
Са скорр., ммоль/л	25	2,61 [ 2,53; 2,82]	24	2,66 [ 2,575; 2,78]	0,6818¹
Фосфор, ммоль/л	25	0,86 [ 0,73; 0,92]	23	0,93 [ 0,87; 1,04]	0,0036¹
Альбумин, г/л	25	44,6 [ 43,0; 47,0]	24	44,5 [ 43,4; 45,85]	0,6818¹
рСКФ, мл/мин/1,73 м²	25	88 [ 79; 95]	25	82 [ 73; 90]	0,2179¹
25(ОН)D, нг/мл	13	32,4 [ 20,0; 43,2]	6	17,885 [ 12,5; 32,0]	0,1740¹
иПТГ, пг/мл	25	149,5 [ 101,3; 209,2]	25	134,3 [ 110,2; 188,7]	0,8310¹
Са суточной мочи, ммоль/сут	24	8,4975 [ 7,3725; 11,5275]	25	8,26 [ 6,47; 10,71]	0,3524¹
Щелочная фосфатаза, Ед/л	23	66 [ 55; 108]	20	110 [ 83; 128]	0,0144¹
Остеокальцин, нг/мл	21	35,77 [ 21,60; 46,05]	17	69,37 [ 52,33; 77,87]	0,0017¹
Бета-кросслапс, нг/мл	21	0,531 [ 0,305; 0,976]	18	1,315 [ 1,010; 1,720]	0,0003¹
Общая характеристика костных нарушений до операции
Остеопороз (на основании результатов DXA и/или наличии НЭП)	25	23 (92%)	25	22 (88%)	1,0000²
Компрессионные переломы	25	3 (12%)	25	5 (20%)	0,7019²
Внепозвоночные остеопоротические переломы	25	4 (16%)	25	4 (16%)	1,0000²
Фиброзно-кистозный остеит	25	2 (8%)	25	1 (4%)	1,0000²
Нефрокальциноз/нефролитиаз	25	17 (68%)	25	16 (64%)	1,0000²
Данные лабораторного обследования после операции
Са общий, ммоль/л	24	2,21 [ 2,175; 2,355]	25	2,27 [ 2,19; 2,34]	0,7872¹
Са ион., ммоль/л	24	1,12 [ 1,08; 1,18]	25	1,11 [ 1,09; 1,20]	0,7039¹
Са скорр., ммоль/л	4	2,20 [ 2,095; 2,245]	1	2,27	-
иПТГ, пг/мл	25	30,35 [ 12,90; 43,02]	25	32,28 [ 22,43; 50,93]	0,2859¹
Развитие нового НЭП после операции
Новый подтвержденный перелом в течение года после операции	13	1 (8%)	14	1 (7%)	1,0000²

**Table:** Таблица 2. Сравнительная характеристика групп по результатам DXA до и после операции Сокращения: МПК — минеральная плотность костной ткани. Примечания: 1 — критерий Манна-Уитни; 3 — критерий Фримена-Холтона. Поправка Бонферрони: р0 = 0,05/64 = 0,0008.

Признак	Пациенты, принимающие бисфосфонаты перед операцией	Пациенты, не принимающие бисфосфонаты перед операцией	р
N	Me [ Q1; Q3] / n (%)	N	Me [ Q1; Q3] / n (%)
Данные DXA до операции
Минимальное значение Т-критерия	23	-3,3 [ -4,5; -2,7]	23	-3,5 [ -4,4; -2,8]	0,9912¹
Минимальное значение Z-критерия	2	-3,3; -2,0	2	-3,8; -1,9	-
BMD	BMD L1-L4	24	0,913 [ 0,8495; 0,953]	25	0,873 [ 0,842; 0,984]	0,4777¹
BMD total hip	25	0,812 [ 0,775; 0,844]	25	0,855 [ 0,749; 0,898]	0,3130¹
BMD Neck	25	0,772 [ 0,718; 0,816]	25	0,800 [ 0,722; 0,836]	0,2072¹
BMD Radius Total	25	0,478 [ 0,411; 0,530]	24	0,481 [ 0,407; 0,562]	0,6101¹
BMD Radius 33%	25	0,619 [ 0,527; 0,651]	24	0,619 [ 0,534; 0,710]	0,6672¹
Данные DXA после операции
Минимальное значение Т-критерия	23	-3,1 [ -4,4; -2,6]	23	-3 [ -4,4; -2,5]	0,7417¹
Минимальное значение Z-критерия	2	-3,0; -1,3	2	-3,2; -1,6	-
BMD	BMD L1-L4	24	0,9535 [ 0,877; 1,004]	25	0,956 [ 0,892; 1,075]	0,8729¹
BMD total hip	25	0,843 [ 0,788; 0,878]	25	0,882 [ 0,790; 0,943]	0,0952¹
BMD Neck	25	0,778 [ 0,754; 0,817]	25	0,816 [ 0,749; 0,876]	0,1377¹
BMD Radius Total	24	0,4905 [ 0,424; 0,521]	24	0,4975 [ 0,416; 0,5645]	0,8934¹
BMD Radius 33%	24	0,619 [ 0,5595; 0,665]	24	0,6165 [ 0,546; 0,7005]	0,7571¹
Прирост BMD	Прирост BMD L1-L4, %	24	5,4 [ 2,4; 7,65]	25	6,1 [ 3,0; 10,1]	0,3371¹
Прирост BMD total hip, %	25	3,8 [ -0,4; 9,0]	25	4,4 [ 1,3; 8,5]	0,4849¹
Прирост BMD Neck, %	25	1,5 [ -1,2; 5,3]	25	3,2 [ 1,9; 7,6]	0,1903¹
Прирост BMD Radius Total, %	24	2,35 [ -0,8; 7,55]	23	1,8 [ -1,7; 3,5]	0,5371¹
Прирост BMD Radius 33%, %	24	2,45 [ -1,45; 6,4]	23	3,2 [ -2,1; 6,0]	0,8900¹

Дополнительно мы проанализировали показатели кальций-фосфорного обмена в раннем послеоперационном периоде, статистически значимых различий между группами не выявлено.

При сопоставлении групп по параметрам 3D-DXA на дооперационном этапе статистически значимых различий также не определялось.

Для оценки темпов восстановления костной ткани на фоне стойкой ремиссии ПГПТ нами был рассчитан абсолютный прирост МПК по результатам DXA в основных отделах скелета и 3D-DXA в бедренной кости. В обеих группах показатели были сопоставимы. Изменения на уровне статистической тенденции были получены для параметров 3D-DXA, итоговые абсолютные значения которых были несколько выше во второй группе. Так, выше были показатели толщины кортикального слоя в бедренной кости в целом (1,78 [ 1,71; 1,84] мм против 1,90 [ 1,80; 1,96] мм, р=0,0062) и в ее шейке (1,50 [ 1,40; 1,62] мм против 1,63 [ 1,52; 1,70] мм, р=0,0130), более подробно результаты представлены в таблице 3. Пример визуализации динамических изменений МПК при 3D-DXA представлен на рис. 2.

**Table:** Таблица 3. Сравнительная характеристика групп по результатам 3D-DXA до и после операции Примечания: 1 — критерий Манна-Уитни. Поправка Бонферрони: р0 = 0,05/64 = 0,0008.

Признак	Пациенты, принимающие бисфосфонаты перед операцией	Пациенты, не принимающие бисфосфонаты перед операцией	р
N	Me [ Q1; Q3] / n (%)	N	Me [ Q1; Q3] / n (%)
Данные 3D-DXA до операции
Минеральная плотность поверхности кортикальной кости TH (мг/см²)	25	132,01 [ 121,84; 141,21]	25	139,13 [ 118,30; 149,55]	0,5094¹
Минеральная плотность поверхности кортикальной кости FN (мг/см²)	25	110,02 [ 94,31; 115,28]	25	116,77 [ 104,03; 124,89]	0,1352¹
Объем минеральной плотности трабекулярной кости TH (мг/см³)	25	120,02 [ 94,73; 132,14]	25	126,61 [ 107,36; 147,17]	0,1567¹
Объем минеральной плотности трабекулярной кости FN (мг/см³)	25	169,42 [ 157,69; 175,44]	25	170,94 [ 134,67; 188,07]	0,7562¹
Объем минеральной плотности кортикальной кости TH (мг/см³)	25	728,55 [ 705,80; 763,64]	25	730,46 [ 683,86; 777,71]	0,9690¹
Объем минеральной плотности кортикальной кости FN (мг/см³)	25	727,00 [ 693,61; 757,71]	25	739,69 [ 688,79; 775,12]	0,6695¹
Толщина кортикальной кости с ТН (мм)	25	1,78 [ 1,74; 1,85]	25	1,84 [ 1,76; 1,94]	0,1352¹
Толщина кортикальной кости в FN (мм)	25	1,51 [ 1,43; 1,59]	25	1,59 [ 1,49; 1,69]	0,0523¹
Данные 3D-DXA после операции
Минеральная плотность поверхности кортикальной кости TH (мг/см²)	25	135,94 [ 123,15; 147,07]	25	140,49 [ 131,86; 157,12]	0,1683¹
Минеральная плотность поверхности кортикальной кости FN (мг/см²)	25	106,87 [ 103,72; 113,95]	25	121,18 [ 106,24; 125,63]	0,0572¹
Объем минеральной плотности трабекулярной кости TH (мг/см³)	25	132,59 [ 106,23; 143,80]	25	139,57 [ 126,39; 161,94]	0,1073¹
Объем минеральной плотности трабекулярной кости FN (мг/см³)	25	177,37 [ 159,89; 192,46]	25	184,81 [ 153,66; 201,67]	0,3320¹
Объем минеральной плотности кортикальной кости TH (мг/см³)	25	754,30 [ 714,50; 783,87]	25	731,65 [ 701,05; 786,30]	0,7562¹
Объем минеральной плотности кортикальной кости FN (мг/см³)	25	747,08 [ 700,96; 768,78]	25	732,91 [ 698,32; 789,36]	0,8462¹
Толщина кортикальной кости с ТН (мм)	25	1,78 [ 1,71; 1,84]	25	1,90 [ 1,80; 1,96]	0,0062¹
Толщина кортикальной кости в FN (мм)	25	1,50 [ 1,40; 1,62]	25	1,63 [ 1,52; 1,70]	0,0130¹

**Figure:**
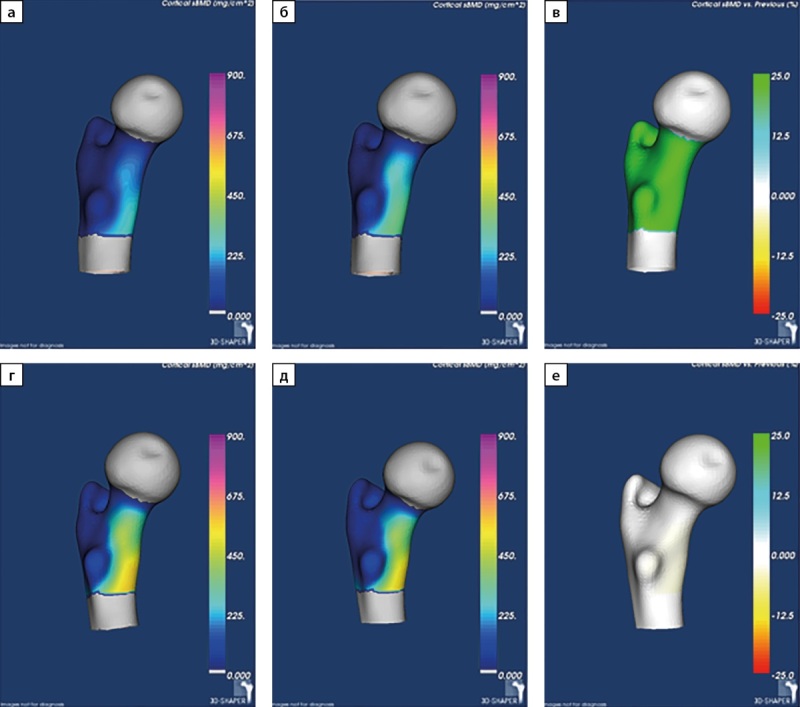
Рисунок 2. Пример визуализации минеральной плотности поверхности кортикальной кости в динамике, полученные с помощью программного обеспечения 3D-Shaper. Примечание: а)-в) пациент из группы 1; г)-е) пациент из группы 2.а), г) визуализация минеральной плотности поверхности кортикальной кости дорсального отдела бедренной кости до ПТЭ (мг/см²); б), д) визуализация минеральной плотности поверхности кортикальной кости дорсального отдела бедренной кости после ПТЭ (мг/см²); в), е) визуализация изменения минеральной плотности поверхности кортикальной кости дорсального отдела бедренной кости до ПТЭ и после ПТЭ; изменение минеральной плотности поверхности кортикальной кости показано в виде ее прироста после ПТЭ относительно бывшей минеральной плотности поверхности кортикальной кости до ПТЭ (%).

Компрессионные переломы в послеоперационном периоде произошли у одного пациента (1/13, 8%) из группы 1 и у одного из группы 2 (1/14, 7%).

Далее мы провели анализ послеоперационных изменений внутри каждой группы. При сравнении результатов DXA у пациентов, получавших БФ до ПТЭ (табл. 4), статистически значимые различия по абсолютным значениям МПК были получены только в поясничном отделе позвоночника (0,913 [ 0,8495; 0,935] против 0,9535 [ 0,877; 1,004], р<0,001) (рис. 3), изменения в бедренной и лучевой костях находились на уровне статистической тенденции (табл. 4). По данным 3D-DXA, статистически значимые различия были выявлены лишь по объему минеральной плотности трабекулярной кости в бедре в целом (120,02 [ 94,73; 132,14] мг/см³ против 132,59 [ 106,23; 143,80] мг/см³, р=0,001) (рис. 4).

**Table:** Таблица 4. Динамическая характеристика состояния костной ткани в группе 1 после ПТЭ Сокращения: иПТГ — интактный паратгормон. Примечание: поправка Бонферрони: р0 = 0,05/13 = 0,0038.

Признак	До операции	После операции	p, W
N	Me [ Q1; Q3]	N	Me [ Q1; Q3]
Данные DXA
BMD	BMD L1-L4	24	0,913 [ 0,8495; 0,953]	24	0,9535 [ 0,877; 1,004]	<0,001
BMD total hip	25	0,812 [ 0,775; 0,844]	25	0,843 [ 0,788; 0,878]	0,008
BMD Neck	25	0,772 [ 0,718; 0,816]	25	0,778 [ 0,754; 0,817]	0,044
BMD Radius Total	25	0,478 [ 0,411; 0,530]	24	0,4905 [ 0,424; 0,521]	0,019
BMD Radius 33%	25	0,619 [ 0,527; 0,651]	24	0,619 [ 0,5595; 0,665]	0,023
Данные 3D-DXA
Минеральная плотность поверхности кортикальной кости TH (мг/см²)	25	132,01 [ 121,84; 141,21]	25	135,94 [ 123,15; 147,07]	0,035
Минеральная плотность поверхности кортикальной кости FN (мг/см²)	25	110,02 [ 94,31; 115,28]	25	106,87 [ 103,72; 113,95]	0,367
Объем минеральной плотности трабекулярной кости TH (мг/см³)	25	120,02 [ 94,73; 132,14]	25	132,59 [ 106,23; 143,80]	0,001
Объем минеральной плотности трабекулярной кости FN (мг/см³)	25	169,42 [ 157,69; 175,44]	25	177,37 [ 159,89; 192,46]	0,010
Объем минеральной плотности кортикальной кости TH (мг/см³)	25	728,55 [ 705,80; 763,64]	25	754,30 [ 714,50; 783,87]	0,069
Объем минеральной плотности кортикальной кости FN (мг/см³)	25	727,00 [ 693,61; 757,71]	25	747,08 [ 700,96; 768,78]	0,040
Толщина кортикальной кости с ТН (мм)	25	1,78 [ 1,74; 1,85]	25	1,78 [ 1,71; 1,84]	0,581
Толщина кортикальной кости в FN (мм)	25	1,51 [ 1,43; 1,59]	25	1,50 [ 1,40; 1,62]	0,459

**Figure:**
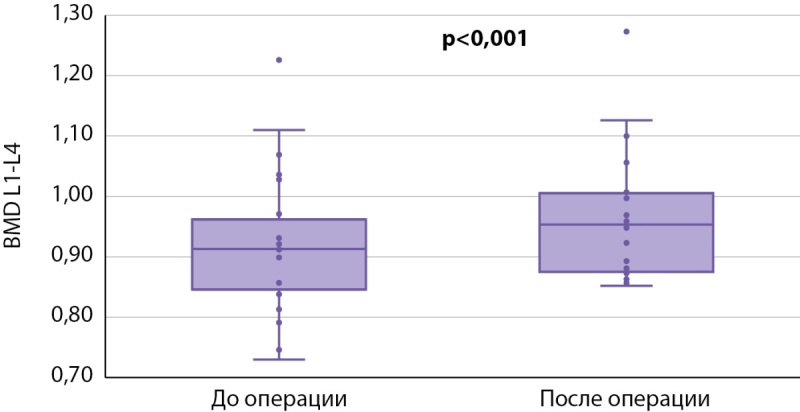
Рисунок 3. Признаки DXA, по которым обнаружены статистически значимые различия в динамике в группе 1.

**Figure:**
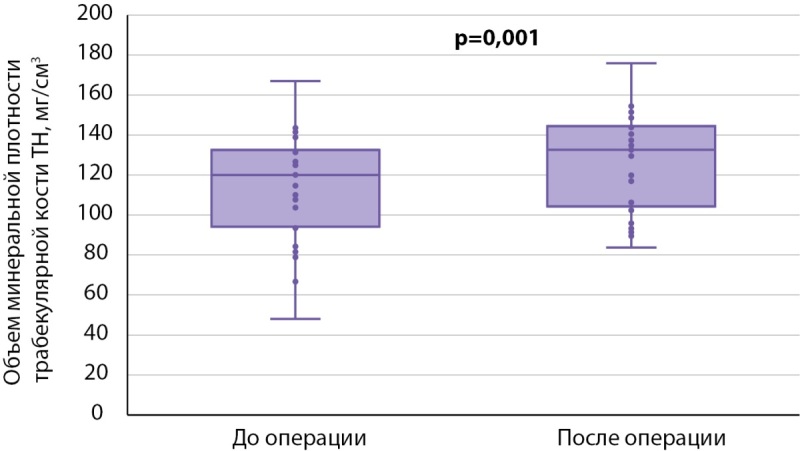
Рисунок 4. Признаки 3D-DXA, по которым обнаружены статистически значимые различия в динамике в группе 1.

При анализе до- и послеоперационных результатов DXA во второй группе (табл. 5) статистически значимые различия по абсолютным показателям МПК наблюдались в поясничном отделе (0,873 [ 0,842; 0,984] против 0,956 [ 0,892; 1,075], р<0,001), в бедре в целом (0,855 [ 0,749; 0,898] против 0,882 [ 0,790; 0,943], р<0,001) и его шейке (0,800 [ 0,722; 0,836] против 0,816 [ 0,749; 0,876], р=0,001) (рис. 5).

**Table:** Таблица 5. Сравнение групп пациентов, не принимающих медикаментозную терапию перед операцией, в динамике Сокращения: иПТГ — интактный паратгормон. Примечание: поправка Бонферрони: р0 = 0,05/13 = 0,0038.

Признак	До операции	После операции	p, W
N	Me [ Q1; Q3]	N	Me [ Q1; Q3]
Данные DXA
BMD	BMD L1-L4	25	0,873 [ 0,842; 0,984]	25	0,956 [ 0,892; 1,075]	<0,001
BMD total hip	25	0,855 [ 0,749; 0,898]	25	0,882 [ 0,790; 0,943]	<0,001
BMD Neck	25	0,800 [ 0,722; 0,836]	25	0,816 [ 0,749; 0,876]	0,001
BMD Radius Total	23	0,479 [ 0,407; 0,562]	23	0,499 [ 0,411; 0,565]	0,089
BMD Radius 33%	23	0,608 [ 0,533; 0,699]	23	0,634 [ 0,524; 0,704]	0,053
Данные 3D-DXA
Минеральная плотность поверхности кортикальной кости TH (мг/см²)	25	139,13 [ 118,30; 149,55]	25	140,49 [ 131,86; 157,12]	0,001
Минеральная плотность поверхности кортикальной кости FN (мг/см²)	25	116,77 [ 104,03; 124,89]	25	121,18 [ 106,24; 125,63]	0,021
Объем минеральной плотности трабекулярной кости TH (мг/см³)	25	126,61 [ 107,36; 147,17]	25	139,57 [ 126,39; 161,94]	<0,001
Объем минеральной плотности трабекулярной кости FN (мг/см³)	25	170,94 [ 134,67; 188,07]	25	184,81 [ 153,66; 201,67]	<0,001
Объем минеральной плотности кортикальной кости TH (мг/см³)	25	730,46 [ 683,86; 777,71]	25	731,65 [ 701,05; 786,30]	0,051
Объем минеральной плотности кортикальной кости FN (мг/см³)	25	739,69 [ 688,79; 775,12]	25	732,91 [ 698,32; 789,36]	0,135
Толщина кортикальной кости с ТН (мм)	25	1,84 [ 1,76; 1,94]	25	1,90 [ 1,80; 1,96]	0,009
Толщина кортикальной кости в FN (мм)	25	1,59 [ 1,49; 1,69]	25	1,63 [ 1,52; 1,70]	0,221

**Figure:**
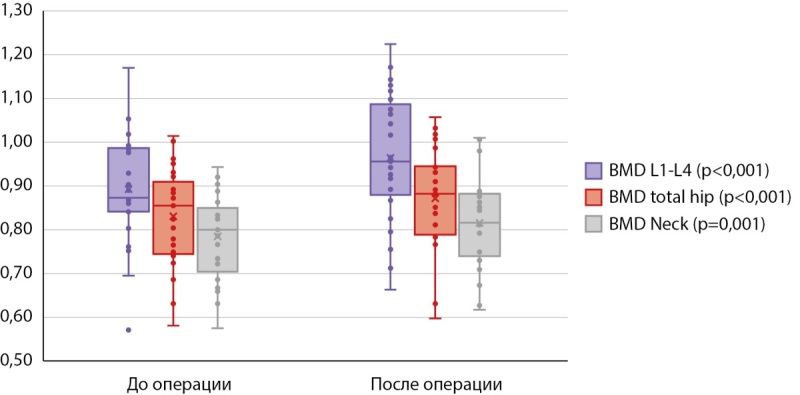
Рисунок 5. Признаки DXA, по которым обнаружены статистически значимые различия в динамике в группе 2.

По данным 3D-DXA, статистически значимые различия были выявлены по трем из восьми проанализированных показателей. Здесь так же, как и в случае с группой пациентов, принимающих бисфосфонаты в дооперационном периоде, увеличились объем минеральной плотности трабекулярной кости в бедре в целом (126,32 [ 107,36; 147,17] мг/см³ против 139,57 [ 126,39; 161,94] мг/см³, р<0,001) и его шейке (170,94 [ 134,67; 188,07] мг/см³ против 184,81 [ 153,66; 201,67] мг/см³, р<0,001) (рис. 6). Статистически значимые различия дополнительно наблюдались по минеральной плотности поверхности кортикальной кости в бедре в целом (139,13 [ 118,30; 149,55] мг/см² против 140,49 [ 131,86; 157,12] мг/см², р=0,001).

**Figure:**
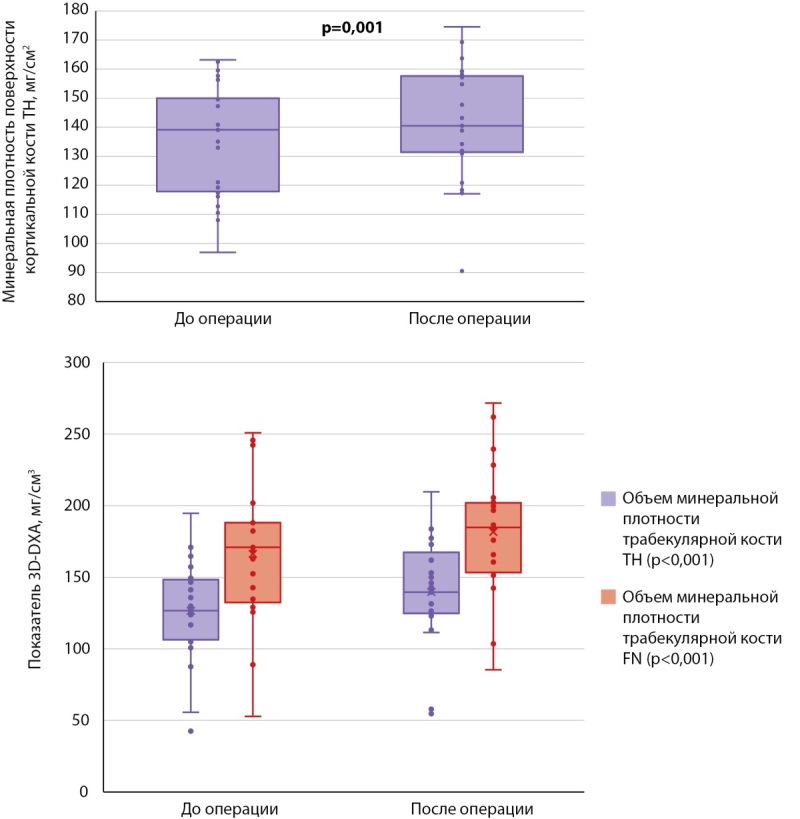
Рисунок 6. Признаки 3D-DXA, по которым обнаружены статистически значимые различия в динамике в группе 2.

## ОБСУЖДЕНИЕ

## Репрезентативность выборок

Набор участников проводился только в ФГБУ «НМИЦ эндокринологии» Минздрава России — экспертном учреждении, в которое направляются пациенты с тяжелым и/или нетипичным течением заболевания, что ограничивает репрезентативность полученной выборки.

## Сопоставление с другими публикациями

Единственным патогенетическим методом лечения ПГПТ остается ПТЭ, при этом наличие костных осложнений заболевания в виде снижения МПК и/или наличия НЭП вынесено экспертами в качестве самостоятельного абсолютного показания к операции [5]. Многочисленные исследования подтверждают непосредственное положительное влияние удаления измененных ОЩЖ на состояние костной ткани без применения дополнительной антиостеопоротической терапии. Быстрый прирост МПК в проксимальном отделе бедренной кости и поясничном отделе позвоночника наблюдается в течение первых 1–2 лет после операции, кроме того, улучшаются значения трабекулярного костного индекса, отражающего микроархитектонику костной ткани [8][9]. Важно отметить, что данные изменения определяются и у пациентов старшей возрастной группы (>75 лет) [10]. Дальнейший прирост МПК может сохраняться на протяжении достаточно длительного периода времени (10–15 лет после ПТЭ), достигая +8% в поясничном отделе позвоночника и +5% в шейке бедренной кости относительно исходных значений. Помимо влияния на МПК, хирургическое лечение ассоциировано с достоверным снижением риска переломов. По результатам метаанализа Kongsaree N. и соавт. (73 778 человек в группе ПТЭ против 164 410 человек в группе наблюдения), хирургическое лечение приводило к достоверному снижению риска перелома в любом отделе скелета в целом (ОР 0,80; 95% ДИ, 0,74–0,86), и — отдельно — в бедренной кости (ОР, 0,63; 95% ДИ, 0,52–0,76) [11].

Антиостеопоротическая терапия у пациентов с ПГПТ остается важным инструментом для дооперационной коррекции гиперкальциемии, а также улучшает состояние костной ткани среди лиц, для которых операция должна быть отложена или не может быть проведена ввиду высоких периоперационных рисков [5]. В ряде работ продемонстрировано положительное влияние консервативной терапии (БФ и деносумаба) на МПК, а также на показатели кальций-фосфорного обмена и маркеров костного ремоделирования при бессимптомных формах ПГПТ или при невозможности проведения ПТЭ. В то же время снижения частоты новых переломов, как основного критерия эффективности антиостеопоротической терапии, не отмечалось [12][13].

Исследования по оценке комбинированного медикаментозного и хирургического лечения костных нарушений у пациентов с ПГПТ лимитированы, хотя данный вопрос крайне актуален для российской популяции. По результатам анализа Базы данных клинико-эпидемиологического мониторинга ПГПТ, в РФ примерно 1/3 пациентов (28,7%) принимают или принимали бисфосфонаты в различных режимах (преимущественно алендронат 70 мг 1 раз в неделю) [4].

Может ли данная терапия сказаться на восстановлении костной ткани после операции — открытый вопрос, требующий дополнительных исследований. Бисфосфонаты эффективно подавляют избыточную костную резорбцию благодаря высокой аффинности к кристаллам гидроксиапатита, преимущественно в местах активного ремоделирования кости, но не влияют на костный синтез. За счет такого механизма действия данные препараты способны длительно находиться в костном депо (до 10 лет). При постменопаузальном остеопорозе подавление избыточной активности остеокластов имеет преимущества, так как костное ремоделирование становится более сбалансированным [14]. Однако при ПГПТ патогенез костных нарушений иной, что в целом связано с высоким костным обменом на фоне избыточной гиперсекреции ПТГ. После ПТЭ секреция ПТГ возвращается к физиологическому «пульсовому» режиму, стимулирующему как костеобразование, так и костную резорбцию. В послеоперационном периоде оба процесса крайне важны для удаления дефектной костной ткани и замещения ее новой с достаточными прочностью и упругостью [15]. Можно предположить, что назначение БФ может мешать активному костеобразованию. Так, в исследовании PaTH, в котором изучалась эффективность рекомбинантного ПТГ в моно- или комбинированной терапии с бисфосфонатами среди пациентов с постменопаузальным остеопорозом, было выявлено значительное увеличение образования костной ткани именно в первой группе. Авторы пришли к выводу, что комбинированная терапия подавляет анаболическое влияние аналога ПТГ на костную ткань [16].

Важной характеристикой нашего исследования является сопоставимость групп по исходным значениям МПК накануне операции. Это позволило нивелировать влияние степени выраженности костных осложнений ПГПТ до операции на темпы прироста МПК после. При сравнении абсолютных показателей и процента прироста МПК в различных отделах скелета до и после ПТЭ группы оказались сопоставимы, что подтверждает положительные изменения после операции в обеих группах. В целом, это не противоречит данным Orr L.E. и соавт. [7]. Авторы провели ретроспективное когортное исследование 1737 пациентов с первичным гиперпаратиреозом, которые были разделены на 5 групп в зависимости от проводимой терапии: наблюдение, монотерапия бисфосфонатами, ПТЭ, бисфосфонаты, затем ПТЭ; ПТЭ, после бисфосфонаты. В отличие от нашего исследования сравнительный анализ по приросту МПК проводился только между группами ПТЭ и ПТЭ далее БФ, что прежде всего было связано с нехваткой данных DXA. Достоверных различий не наблюдалось, изменения МПК в бедренной кости составили +5,50%, (95% ДИ 3,39; 7,61) и + 6,30% (95% ДИ 2,53; 10,07) соответственно. Chor HJ. и соавт. также сопоставили эффективность комбинированного лечения (ПТЭ и затем БФ) по сравнению с изолированной ПТЭ. Но в отличие от предыдущей работы, Chor HJ. и cоавт. был сделан вывод, что у пациентов с остеопорозом на комбинированной терапии нет преимуществ по сравнению с пациентами, не принимавшими БФ, и напротив, данная комбинация могла препятствовать увеличению костной массы в послеоперационном периоде [17].

Важно отметить, что показатели МПК все же остаются суррогатным критерием при оценке эффективности антиостеопротической терапии. Основным критерием остается отсутствие новых НЭП. Несмотря на положительные изменения МПК, новые компрессионные переломы произошли в обеих группах, при этом частота их развития была сопоставима. Вероятно, к основной причине новых НЭП можно отнести исходно значимые изменения в костной ткани до операции и недостаточный период восстановления после нее. В исследовании Orr L.E. и соавт. ПТЭ, а также прием бисфосфонатов с последующей ПТЭ были ассоциированы со снижением риска PTX (ОР 0,55, 95% ДИ 0,35; 0,84 и ОР 0,46, 95% ДИ 0,25; 0,83 соответственно), что указывает на то, что предшествующая медикаментозная терапия не исключала положительного влияния операции. Напротив, прием БФ, инициированный после ПТЭ, не имел преимуществ в снижении риска НЭП. И более того, авторы пришли к выводу о том, что назначение БФ после операции может нивелировать положительное влияние ПТЭ на риск переломов [7]. Однако, с учетом фармакокинетики препаратов, их способности длительно сохраняться в костном депо, и таким образом влиять на ремоделирование, «временное» деление на до и после ПТЭ может быть условным.

В пользу наибольшего влияния именно операции на риск переломов выступают результаты метаанализа Kongsaree N. и соавт., включающем более 230 тыс. пациентов с ПГПТ (73 778 человек в группе ПТЭ против 164 410 человек в группе наблюдения). ПТЭ ассоциировалась с достоверным снижением риска перелома в любом отделе скелета (ОР 0,80; 95% ДИ, 0,74–0,86), а также отдельно перелома бедренной кости (ОР, 0,63; 95% ДИ, 0,52–0,76). При этом снижения риска переломов костей предплечья и позвонков не наблюдалось (переломы у 3574 и 3795 пациентов соответственно) [11].

Исследования с применением технологии 3D-DXA при ПГПТ пока немногочисленны, и в основном касались сравнительного анализа минеральных характеристик кортикального и трабекулярного компонентов между пациентами с ПГПТ и здоровыми добровольцами, а также лицами с постменопаузальным остеопорозом. Пациенты с ПГПТ ожидаемо характеризовались худшими значениями поверхностной минеральной плотности и толщины кортикальной ткани, различия по общей и трабекулярной объемной плотности были продемонстрированы только при сопоставлении с группой постменопаузального остеопороза [18][19]. Таким образом, устранение избытка ПТГ в первую очередь должно сказаться на состоянии именно кортикального компонента. В нашем исследовании у пациентов без предшествующей антиостеопоротической терапии после ПТЭ на уровне статистической тенденции отмечались более высокие значения толщины кортикального слоя в бедренной кости в целом и в его шейке, что потенциально указывает на негативное влияние бисфосфонатов на восстановление кортикального компонента после операции. Данную гипотезу поддерживают результаты, полученные нами при сопоставлении как абсолютных значений МПК, так и объемных и поверхностных характеристик внутри группы. Для пациентов с ПТЭ без приема БФ в анамнезе статистически значимые различия были обнаружены по большему числу признаков. При этом они затрагивали как абсолютные значения МПК (в поясничном отделе позвоночника, бедре в целом и шейке), так и параметры 3D-DXA (объем минеральной плотности трабекулярной кости в бедре в целом и его шейке, минеральной плотности поверхности кортикальной кости в бедре в целом).

## ЗАКЛЮЧЕНИЕ

Метод 3D-DXA позволяет получить объемные данные о костной структуре, что помогает более точно оценить не только минеральную плотность, но и микроархитектонику костной ткани. Кроме того, данный метод может помочь в оценке функционального состояния костей и предсказании риска переломов, что крайне важно для пациентов с ПГПТ. Учитывая частое назначение бисфосфонатов накануне ПТЭ, актуальным остается вопрос о влиянии данной терапии на послеоперационное течение заболевания. Некоторые исследования сосредоточились на возможности назначения бисфосфонатов после хирургического вмешательства, однако основные выводы указывают на отсутствие преимуществ такого подхода и, более того, на потенциально негативное влияние на риск переломов. В настоящем исследовании впервые был проведен детальный анализ влияния предоперационной терапии бисфосфонатами на динамику показателей МПК у пациентов с ПГПТ через год после паратиреоидэктомии с использованием как DXA, так и 3D-DXA. Согласно полученным результатам восстановление МПК в группе бисфосфонатов в комбинации с ПТЭ по сравнению с изолированным хирургическим лечением протекало хуже, особенно в отношении кортикальной костной ткани. Требуются дальнейшие исследования для подтверждения полученных данных.

## Направления дальнейших исследований

В дальнейшем планируется расширение выборки пациентов и увеличение срока наблюдения за пациентами.

## ДОПОЛНИТЕЛЬНАЯ ИНФОРМАЦИЯ

Источники финансирования. Работа выполнена по инициативе авторов без привлечения финансирования.

Конфликт интересов. Авторы декларируют отсутствие явных и потенциальных конфликтов интересов, связанных с содержанием настоящей статьи

Участие авторов. Першина-Милютина А.П. — существенный вклад в концепцию исследования, в получение, анализ данных и интерпретацию результатов, написание статьи; Еремкина А.К. — существенный вклад в дизайн исследования, в получение данных и интерпретацию результатов, написание статьи; Ожималов И.Д. — существенный вклад в получение данных, интерпретацию результатов, написание статьи; Хайриева А.В.— существенный вклад в получение данных, интерпретацию результатов; Горбачева А.М. — существенный вклад в концепцию исследования, интерпретацию результатов; Ронжина С.В. — существенный вклад в получение данных, интерпретацию результатов; Мокрышева Н.Г. — внесение в рукопись существенной (важной) правки с целью повышения научной ценности статьи.

Все авторы одобрили финальную версию статьи перед публикацией, выразили согласие нести ответственность за все аспекты работы, подразумевающую надлежащее изучение и решение вопросов, связанных с точностью или добросовестностью любой части работы.
